# Numerical analysis on the effects of microfluidic-based bioprinting parameters on the microfiber geometrical outcomes

**DOI:** 10.1038/s41598-022-07392-0

**Published:** 2022-03-01

**Authors:** Ahmadreza Zaeri, Ralf Zgeib, Kai Cao, Fucheng Zhang, Robert C. Chang

**Affiliations:** Department of Mechanical Engineering, Stevens Institute of Technology, Hoboken, NJ 07030 USA

**Keywords:** Biomedical engineering, Design, synthesis and processing

## Abstract

The application of microfluidics technology in additive manufacturing is an emerging approach that makes possible the fabrication of functional three-dimensional cell-laden structured biomaterials. A key challenge that needs to be addressed using a microfluidic-based printhead (MBP) is increasing the controllability over the properties of the fabricated microtissue. Herein, an MBP platform is numerically simulated for the fabrication of solid and hollow microfibers using a microfluidic channel system with high level of controllability over the microfiber geometrical outcomes. Specifically, the generation of microfibers is enabled by studying the effects of microfluidic-based bioprinting parameters that capture the different range of design, bioink material, and process parameter dependencies as numerically modeled as a multiphysics problem. Furthermore, the numerical model is verified and validated, exhibiting good agreement with literature-derived experimental data in terms of microfiber geometrical outcomes. Additionally, a predictive mathematical formula that correlates the dimensionless process parameters with dimensionless geometrical outcomes is presented to calculate the geometrical outcomes of the microfibers. This formula is expected to be applicable for bioinks within a prescribed range of the density and viscosity value. The MBP applications are highlighted towards precision fabrication of heterogeneous microstructures with functionally graded properties to be used in organ generation, disease modeling, and drug testing studies.

## Introduction

### Introduction to bioprinting

Each year, millions of patients worldwide await organ transplantation due to the limitation of donor organs. Also, disease modeling and drug testing technology platforms necessitate living tissues for high-fidelity experimental workflows that yield physiologically relevant biological insights. In this respect, additive manufacturing (AM) encapsulates 3D printing technologies, including bioprinting towards realizing engineered tissues, organ generation, and disease modeling with the computer-aided precision fabrication of native-like tissue constructs. The characteristic AM attributes enable fast, accurate control over the spatial location of multiple cell types and biomaterials in an automated patterning process both in vitro and in vivo. However, current limitations with AM techniques exist, including printing at high resolutions, along with precise microfiber geometrical outcomes towards functionally graded tissue constructs. To address these issues, the convergence of microfluidic and AM technologies will be advanced to augment the accuracy and control over the realizable geometrical outcomes.

### Microfluidic systems towards microfiber fabrication

In general, microfluidic technology is composed of microchannels that guide, mix, and create a platform for the chemical reaction of various fluids in a high-precision way. The most prominent features of a microfluidic system are low Reynolds number (Re), laminar flow, biocompatibility, flexibility, and micro/nanoscale manipulation of different flows which have encouraged AM practitioners to leverage the attributes of microfluidics towards bioprinting, organ-on-a-chip, and lab-on-a-chip to resemble living tissue microstructures. However, the difficulty of scaling up the tissue structures, handling fragile and wet hydrogel fibers, and the generation of microfibers with desired geometrical outcomes has hampered the adoption of microfluidic systems for manufacturing tissues. To address these bottlenecks, the integration of the microfluidic approach within AM-assisted extrusion bioprinter represents a recent trend that yields layered scale-up of precise tissue constructs^1^. In general, microfluidic systems have been used for the fabrication of microfibers for quite a while. However, the fabricated microfibers have been collected within a container or spun around a rod. In a recent study on hollow microfiber fabrication using a microfluidic system, Yu et al.^1^ fabricated a spatiotemporally controlled spinning of heterogeneous hollow sodium alginate solution and the CaCl_2_ microfibers on a chip based on microfluidic spinning technology. Their technique is cost-effective, simple, and can fabricate fiber with various morphologies and compositions. Polyvinyl Alcohol (PVA) solution is implemented as a core flow owing to its high viscosity, inertness, permeability, and ability to stabilize the core flows during the fiber spinning process. The increase in the flow rate of inner core flow results in an increment in the inner diameter of hollow fiber from 50 to 160 μm and the increase in sample flow rate results in the reduction of inner diameter. Similar microfluidics technology was incorporated to fabricate heterogeneous solid microfibers. In this respect, Kang et al.^2^ fabricated a continuous solid microfiber by mimicking the silk-spinning process of spiders that can digitally tune the material composition and topography of the fibers with a spatially controlled co-culture of encapsulated cells. They used multi-layer cylindrical^3^ and rectangular channels to guide the CaCl_2_ crosslinking sheath flow and the alginate stream from the channel wall.

### Incorporation of microfluidic printhead towards bioprinting

In order to directly write or print the high-precision fabricated microfiber in an ordered pattern toward high-fidelity tissue constructs, the available microfluidic technology is advanced to be integrated into the bioprinting system to address the lack of direct printing of microfibers. From the perspective of microfluidic principles integrated with bioprinting systems, some studies have examined the design and characterization of microfluidic-based printheads for bioprinters for hollow and solid microfibers. Regarding solid microfiber fabrication, Colosi et al. fabricated heterogeneous 3D tissue constructs using microfluidic printhead bioprinting^4^. They used low viscosity bioink to increase print speed, resolution (100 μm), and cell density. Moreover, they used a coaxial needle extrusion system with a mixture of alginate, GelMa, photoinitiator, and cell and achieved a 75% viability. In another study on the fabrication of solid microfibers, Hardin et al. developed a multi-material 3D printing using microfluidic printheads that are able to switch the flow of viscoelastic material during fabrication and enable the fabrication of heterogeneous tissue constructs with controlled compositional and property gradients^5^. This technique is accompanied by high cell viability for different cell types such as cartilage cells^6^. Similarly, Ghorbanian et al. designed a microfluidic direct writer of solid microfibers with an integrated de-clogging mechanism for fabricating cell-laden hydrogel constructs with coaxial streams of cell-laden sodium alginate and calcium chloride solutions^7^. They used Ethylenediaminetetraacetic acid (EDTA) as a de-clogging agent of alginate gel clogs. For this, when the clog is formed, all pumps are stopped and the declogging agent pumped through the channels. Furthermore, Costantini et al. developed a microfluidic-enhanced 3D bioprinting of aligned myoblast-laden hydrogel-based solid microfibers (PEG-fibrinogen solid fibers) coupled to a co-axial needle extruder^8^. Based on the in vitro examination, after 21 days of culture in vitro, the myoblast cells properly spread, fuse, and align long-range multinucleated myotubes, containing abundant functional expressions of myosin heavy chain and laminin. In another study on the solid microfibers, Gao et al. showed that, unlike the outer channel diameter parameter, the inner needle channel diameter can change the diameter of the hollow microfiber^9^. Based on this work, the straight filament is achieved when the stage movement speed is prescribed between 750 mm/min and 1150 mm/min. Their results show that microfiber geometrical outcomes exhibit a parametric dependence on flow rate, viscosity, density, crosslinking, and channel dimension^10^. By modulating these parameters, different fiber diameters can be generated from a couple of microns (16–90 μm^10^) to hundreds of microns.

Regarding the printing of heterogeneous hollow microfibers using the microfluidic-based bioprinting, Pi et al. investigated the digitally tunable microfluidic bioprinting towards the fabrication of multilayered cannular tissues that can be actively perfused with biomolecules to promote cell growth and proliferation^11^. Typically, coaxially printed hollow fibers can be used for emulating many tissues with microtubular structures but are particularly promising in the vascularization of larger scaffolds^12^.

### Numerical studies

There are few numerical studies on droplet formation in the microfluidics system to be used for bioprinting systems such as^13,14^. For example, Khater et al. numerically studied a picolitre agar droplet breakup in microfluidics^15^. The numerical simulations show that ratios of dispersed phase to continuous phase (φ) and the capillary number are the key parameters controlling the agar droplet size and formation regime, from dripping to jetting. Also, Ju et al. used CFD-based modeling of the hydrodynamic production process of polymeric microfibers. Their results show a 40% improvement in the calculation of solid fiber diameter^16^.

### Lack of literature on numerical analysis of microfibers

Based on the current studies, microfluidic technology shows a promising integrative future with AM. Moreover, the ability of the microfluidic system to control process parameters such as flow rate facilitates the fabrication of solid and hollow microfiber in a variety of geometrical dimensions. Therefore, the concept of a microfluidic-based printhead will be capable of printing a functionally graded construct in terms of microfiber geometrical size. To date, there is a lack of numerical studies for systematic analysis and prediction of the geometrical outcomes of microfibers that are generated by a microfluidic-based printhead (MBP).

### The main focus of the paper

To address the current challenges, in this paper, the design and numerical analysis for a coaxial channel system of MBP microfluidic channels in COMSOL® Multiphysics are studied, mapping the effect of printing parameters such as flow rate, viscosity, density, and channel angle on printed structural outcomes. To verify and validate the model, the data exhibits good agreement with the experimental data in the literature in terms of the geometrical dimension of hollow and solid microfiber. Additionally, there is currently no model formulation that exists for predicting geometrical outcomes of the microfiber based on process parameters. Therefore, a predictive mathematical formula is introduced that correlates the dimensionless process parameters to the dimensionless geometrical value of solid and hollow microfiber fabricated using MBP. Thus, using this formulation, an approximate range of the inner diameter, outer diameter, and the wall thickness of the hollow microfiber, and the diameter of solid microfiber will be calculated based on any specific process parameters such as channel size and flow rate. These kinds of analyses can lead to a bottom-up fabrication approach that combines a microfluidic printhead with an extrusion-based ABM. Moreover, it offers an avenue to address the fabrication of precise heterogeneous functionally graded tissue constructs.

## Materials and methods

In this section, the design of a microfluidic-based printhead system is discussed. The design of the bioprinter system starts with the initial design of the microfluidic channels for the MBP. Then, a numerical simulation (computational fluid dynamics (CFD)) is used to model the fluid flow in the microchannel and calculate the flow velocity, the shear rate, and the pressure field. Herein, the investigation is focused on the numerical simulation, characterization, and prediction of the MBP channel system for the fabrication of hollow and solid microfiber.

### Concept MBP system design

Various types of designs can be considered for a microfluidic printhead that is capable of fabricating solid and hollow microfibers. Generally, there are two types of microfluidic system that is used for the fabrication of microfibers in the literature. The chip-based microfluidic system (which can be co-axial^1^)^2^ and the co-axial needle-based nozzle system^4,17^. The main concept of both is similar because these systems are designed to separate two or more fluid-flow from each other in a separate channel (chip-based) or needle (needle-based). Therefore, our microfluidic model simulation can be applied to both configurations. Herein, the proposed MBP system includes all the channels to be inside the printhead so that multiple biomaterials^17^ can be mixed and form a heterogeneous microfiber.

**Figure 1:**
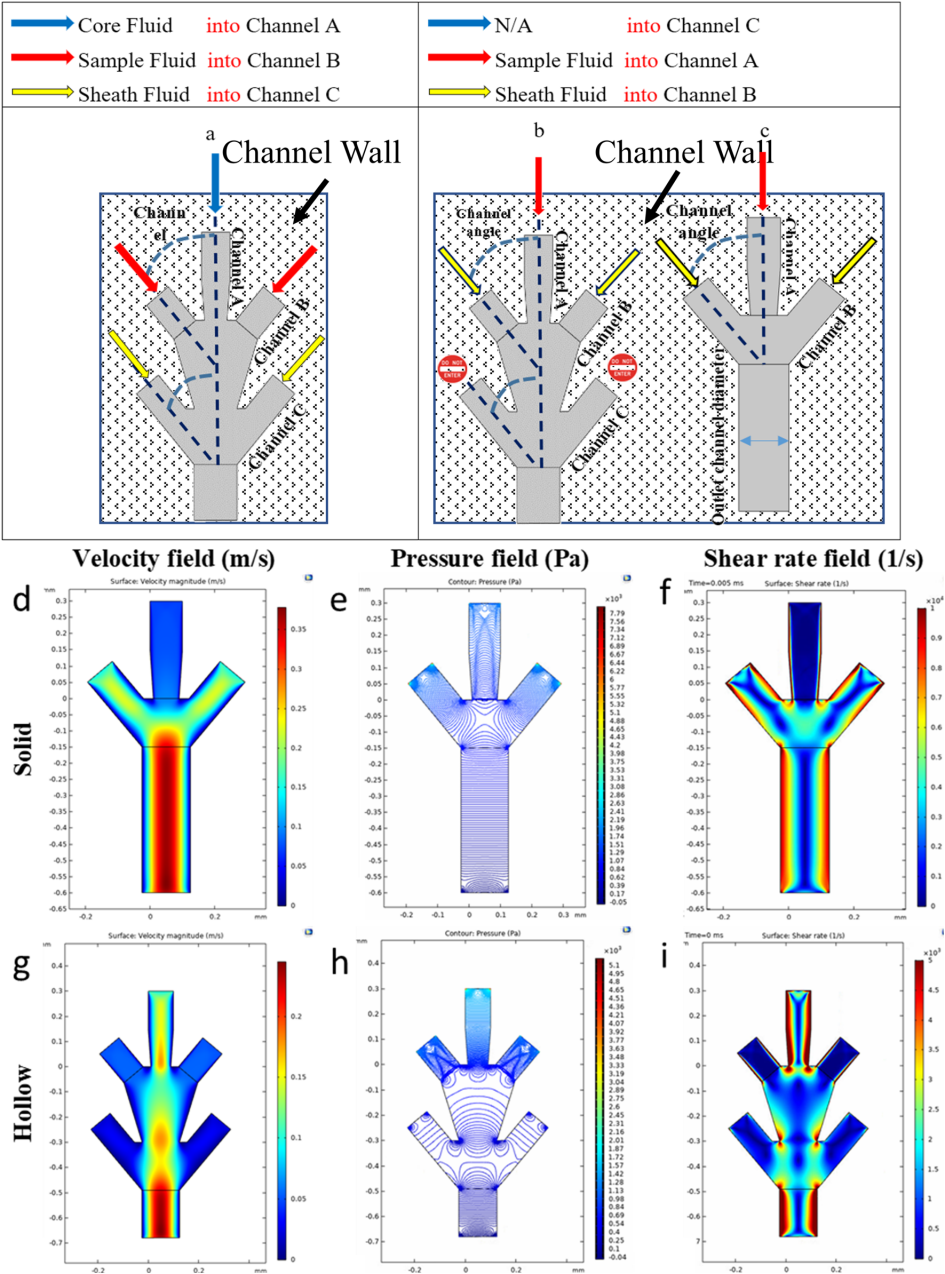
Schematic design of the microfluidic printhead and the numerical modeled parameters. (**a**) The schematic figure of the hollow microfiber fabrication system using the microfluidic channel, (**b**) The schematic figure of the solid microfiber fabrication system using the microfluidic channel. (**d**–**i**) Velocity field, pressure field, and shear rate field in the microfluidic channels embedded within the printhead system, (**d**) Flow velocity field of solid microfiber, (**e**) pressure field of solid microfiber, (**f**) Shear rate field of solid microfiber, (**g**) Flow velocity field of hollow microfiber, (**h**) pressure field of hollow microfiber, (**i**) Shear rate field of hollow microfiber.

Hollow structure features such as large surface area, high permeability, mechanical flexibility, and natural vascular shape, make it a great option for researching the fabrication of the vascular shape structure that mimics native tissues. Such hollow fibers can be fabricated using a multi-phase flow in co-axial channels. The size and configuration of the microfiber can change by manipulating the parameters of concentration, channel size, flow rate, viscosity, polymerization times, and surface coating. The design of the co-axial microfluidic channel which is going to fabricate the hollow and solid microfiber is shown in Fig. 1-a and Fig. 1-b,c, respectively. Also, the channel connection angle was examined by doing a numerical simulation. The fabrication of hollow microfiber (Fig. 1-a) and the solid microfiber is based on the fluid flow in a coaxial channel that includes core flow (either CaCl_2_ crosslinker or a bioink), sample flow (sodium alginate-based bioink), and sheath flow (either CaCl_2_ crosslinker or a lubricant). For the hollow microfiber, the core flow could be either a crosslinking agent (CaCl_2_) or a cell-encapsulated bioink that holds the internal shape of the microfiber. The sheath flow is typically a lubricant or a crosslinker that prevents the hollow fiber to adhere to the channel surface and prevent clogging and cause solidification.

In the case of solid microfiber fabrication (Fig. 1-b,c), the type of fluid flow in the channels is modified. To do so, the sheath channel is blocked to enable the channel system to generate solid microfiber (Fig. 1-b). Then, the sample fluid will flow in channel A and the lubricant fluid or CaCl_2_ crosslinker fluid will flow in channel B. Therefore, only channel A and channel B are recruited for the solid microfiber fabrication system but with different fluids (Fig. 1-c).

Sodium alginate^18^ is one biomaterial that is used for the generation of microfiber. Besides, the CaCl_2_ solution is used as a crosslinker to solidify the bioink. Alginate-based hydrogels are highly advantageous for fabricating cell encapsulated microfibers due to the low cytotoxicity, non-immunogenicity, degradability, mild gelation conditions, easy operability, and definite mechanical strength^19^. For the simulation of hollow microfiber fabrication in the MBP system, the fluid of the sample is a sodium alginate solution. However, the fluid of the sheath and core flow was considered to be CaCl_2_. The non-Newtonian viscosity properties of the alginate were used from reference^20^. For the simulation of the solid microfiber, the channel A fluid was considered to be sodium alginate, and the sheath flow channel was considered to be CaCl_2_.

### Numerical simulation method

First of all the CAD model of the microchannel system is drawn in SolidWorks. Then the model is imported into the COMSOL software. Thereafter, the periphery edges of the model are set to be a no-slip boundary condition. The inlet velocity and flow rate of the bioinks are specified. Then the two-phase flow multiphysics modeling is used to model the whole microchannel system. The same process can be done for most of the microfluidic channel systems to figure out the geometrical feature of the fabricated microfibers.

For the numerical simulation, computational fluid dynamic (CFD) analysis has been carried out to solve continuity (Eq. 2), momentum Navier–Stokes (Eq. 1), and multi-phase flow equation (Eq. 3) in a coupled manner. The governing equations in this problem are nonlinear and the dynamic viscosity model will be non-Newtonian. Since the channel size in the microfluidic chip is very low the Reynolds number is low, so the fluid flow regime is laminar, and the flow of different channels barely mixes by themself. Therefore, based on the following equations the COMSOL® Multiphysics software discretized (triangular mesh) the 2D and 3D geometry of the channel and solve the problem using a time-dependent study. In the Navier–Stokes and continuity equations, ρ is the density (SI unit: kg/m^3^), u is the velocity vector (SI unit: m/s), p is pressure (SI unit: Pa), K is the viscous stress tensor (SI unit: Pa), and F is the volume force vector (SI unit: N/m3)^21^.

1 $$\rho \frac{\partial u}{\partial t}=\nabla \cdot\left[-pI+K\right]+F$$

2 $$\rho \nabla \cdot\mathrm{u}=0$$

In the level set equation for two-phase flow, γ is the reinitialization parameter (set to 1 by default), ε_ls_ is the interface thickness controlling parameter (set to h_max_/2 where h_max_ is the maximum element size in the component), and ϕ can represent any scalar quantity of the flow^21^.

3 $$\frac{\delta \phi }{\delta t}+u\cdot\nabla \phi =\gamma \nabla \cdot\left[{\epsilon }_{ls}\nabla \phi -\phi (1-\phi )\frac{\nabla \phi }{|\nabla \phi |}\right], \phi =phils$$

The wall no-flow conditions were defined as Eq. 4^21^.

4 $$n\cdot\left[{\epsilon }_{ls}\nabla \phi -\phi (1-\phi )\frac{\nabla \phi }{|\nabla \phi |}\right]=0$$

In Eq. 5 to Eq. 7 the non-Newtonian viscosity equations of power-law were defined in the numerical model for sodium alginate (${\mathrm{m}}_{1}=56.53 \left[\mathrm{Pa}.\mathrm{s}\right], {\mathrm{n}}_{1}=0.0863, {\dot{\mathrm{and \gamma }}}_{\mathrm{ref}1}=1\left[\frac{1}{\mathrm{s}}\right]$). Here, ${\dot{{\varvec{\gamma}}}}_{ref1}$ is a lower limit for the evaluation of the shear rate magnitude. The default value for is 10^−2^ s^−1^^21^.

5 $$\rho ={\rho }_{1}+({\rho }_{2}-{\rho }_{1}) \phi $$

6 $$\mu ={\mu }_{1}+({\mu }_{2}-{\mu }_{1}) \phi $$

7 $${\mu }_{1}={m}_{1}{\left(\frac{\dot{\gamma }}{{\dot{\gamma }}_{ref1}}\right)}^{{n}_{1}-1}$$

The definition of Reynold’s number is mentioned in Eq. 8. where U denotes a velocity scale, ${\varvec{\mu}}$ is viscosity, and L denotes a representative length. Reynold’s number is a ratio of inertial and viscous forces. Herein, R is defined as the channel Reynold’s number ratio to be used for the predictive formula of microfiber geometrical outcomes. In Eqs. 9, 10, and 11 two dimensionless number ${{\varvec{R}}}_{{\varvec{A}}}$ and ${{\varvec{R}}}_{{\varvec{B}}}$ which are based on the channel A, B, and C Reynold’s number, were introduced. These dimensionless numbers are used to predict the geometrical values of the fabricated hollow and solid microfiber in the MBP, regardless of any specific process or material parameters.

8 $$Re=\frac{\rho uL}{\mu }=\frac{\mathrm{inertial \,forces }}{\mathrm{viscous \,forces}}$$

9 $${\mathrm{Hollow\, Microfiber}: R}_{A}=\frac{{Re}_{C}+{Re}_{B} }{{Re}_{A}}$$

10 $${\mathrm{Hollow\, Microfiber}: R}_{B}=\frac{{Re}_{C}+{Re}_{A} }{{Re}_{B}}$$

11 $$\mathrm{Solid \,Microfiber}: {R}_{A}=\frac{{Re}_{B} }{{Re}_{A}}$$

Also, the dimensionless geometrical parameters are defined in Eqs. 1–4 for the hollow and solid fabrication microfluidic system, so that any generic MBP system may be able to implement these equations.

1 $$\mathrm{Inner\, diameter\, ratio}=\frac{\mathrm{ Inner \,diameter}}{outlet\, channel\, diameter}$$

2 $$\mathrm{Outer\, diameter\, ratio}=\frac{\mathrm{ Outer \,diameter}}{outlet \,channel\, diameter}$$

3 $$\mathrm{Wall \,thickness\, ratio}=\frac{\mathrm{ Wall\, thickness}}{outlet \,channel \,diameter}$$

4 $$\mathrm{Solid \,microfiber \,diameter\, ratio}=\frac{\mathrm{ Solid \,microfiber\, diameter}}{outlet \,channel\, diameter}$$

### Numerical model validation

It is clear that the numerical CFD model needs to be validated and verified by comparing the numerically derived geometrical outcome of the fabricated microfiber with the experimental data. There are enough experimental data in the literature such as Kang et al.^1^ and Gao et al.^9^ for the hollow and solid microfibers, respectively. Therefore, we decided to proceed with the available experimental data for the verification of our model. In this paper, the calculated geometrical feature of the hollow and solid microfiber that were compared with the experimental results from Kang et al.^1^ and Gao et al.^9^, resulted in the validation and verification of our numerical model. Although there was some difference between the numerical and experimental results which stem from the involvement of more complicated physics in the experiment and are ignored in the numerical model, this difference is not considerable. This validation of our numerical model confirms the accuracy of the model. Thereafter the predictive model was verified by comparing its predictive results with the experimentally derived value of geometrical feature size in reference^22^.

## Results and discussion

### Introduction of the modeling

Herein, fluid flow numerical analyses have been done for a set of interconnected microfluidic channels embedded within a microfluidic printhead system towards the fabrication of hollow or solid microfibers. Specifically, the phase boundary fields and geometrical feature sizes of the printed microfibers are calculated based on key microfluidic flow model inputs of the core, sample, and sheath flow channel. The fabrication of hollow and solid microfiber is controlled by tuning process parameters such as the flow rate of channels A, B, and C flow. Therefore, in the case of the hollow microfiber fabrication, the core flow is responsible to maintain the inner shape of the hollow microfiber and the sheath flow preserves a gap between the channel wall and the sample fluid. In addition, the sheath flow can function as a crosslinking agent (Fig. 1a). On the other hand, the solid microfiber is generated when the sample flow is surrounded by sheath fluid flow (Fig. 1b). Herein, the effect of different design and process parameters such as channel angle, viscosity, density, flow rate, and the dimensionality of microfluidic channels is investigated to clarify their effect on the geometrical feature size of the fabricated microfiber such as fiber inner/outer diameter and thickness of the hollow and solid fabricated microfibers. Moreover, in order to increase the controllability over the continuity of fabricated microfibers, it is essential to understand the flow regime threshold from droplet to continuous flow based on the value of effective process parameters. Further, the fabrication of functionally graded tissue in terms of high-precision microfiber geometrical outcomes (inner/outer diameters and thickness of microfibers), facilitate the fabrication of tissue with a continuous gradient of mechanical and biological properties. Thereafter, a predictive mathematical equation based on dimensionless numbers is presented to predict the inner/outer diameters and thickness of hollow microfiber and the diameter of solid microfiber. This equation is valid for the fluid density and viscosity in the range of those properties of sodium alginate and CaCl_2_, However, it is valid for the different flow rates and channel wide.

### Numerical calculation of flow velocity field, pressure field, and shear rate field

Based on the numerical analyses approach that was used, the velocity field, pressure field, and shear rate field of the two-phase flow were calculated for different flow rates of the core, sample, and sheath fluids. Figure 1d–i shows the calculated velocity field, pressure field, and shear rate field in microfluidic channels embedded within a microfluidic printhead system for solid and hollow microfiber formation. The figure of the velocity field (Fig. 1d, e) shows the highest velocity magnitude in the center of the channel due to the no-slip boundary conditions in the walls. The velocity magnitude reduces from the fluid flow centerline towards the channel walls. In contrast, the closer the fluid to the wall is, the higher the shear rate will be. Also, the shear rate (Fig. 1f, i) in the sharp edges of the nozzle is maximum so the cells in these domains are prone to damage. Further, the behavior of the pressure field (Fig. 1e,h) is shown in this figure. Since the pressure losses in the channel, therefore as the fluid moves towards the end of the channel, the relative pressure is more likely to reduce to the value of outlet pressure of zero. Considering all the information from the flow velocity, pressure, and shear rate, it is crucial to keep the sample fluid flow that contains the biological cells in the center of the interconnected microfluidic channel system in order to prevent any damages to the cells. Therefore, in this paper, the sample fluid in channel A (for solid microfiber fabrication) and B (for hollow microfiber fabrication) is considered, and all of the numerical analysis is performed for this condition.

### Channel angle effect

**Figure 2:**
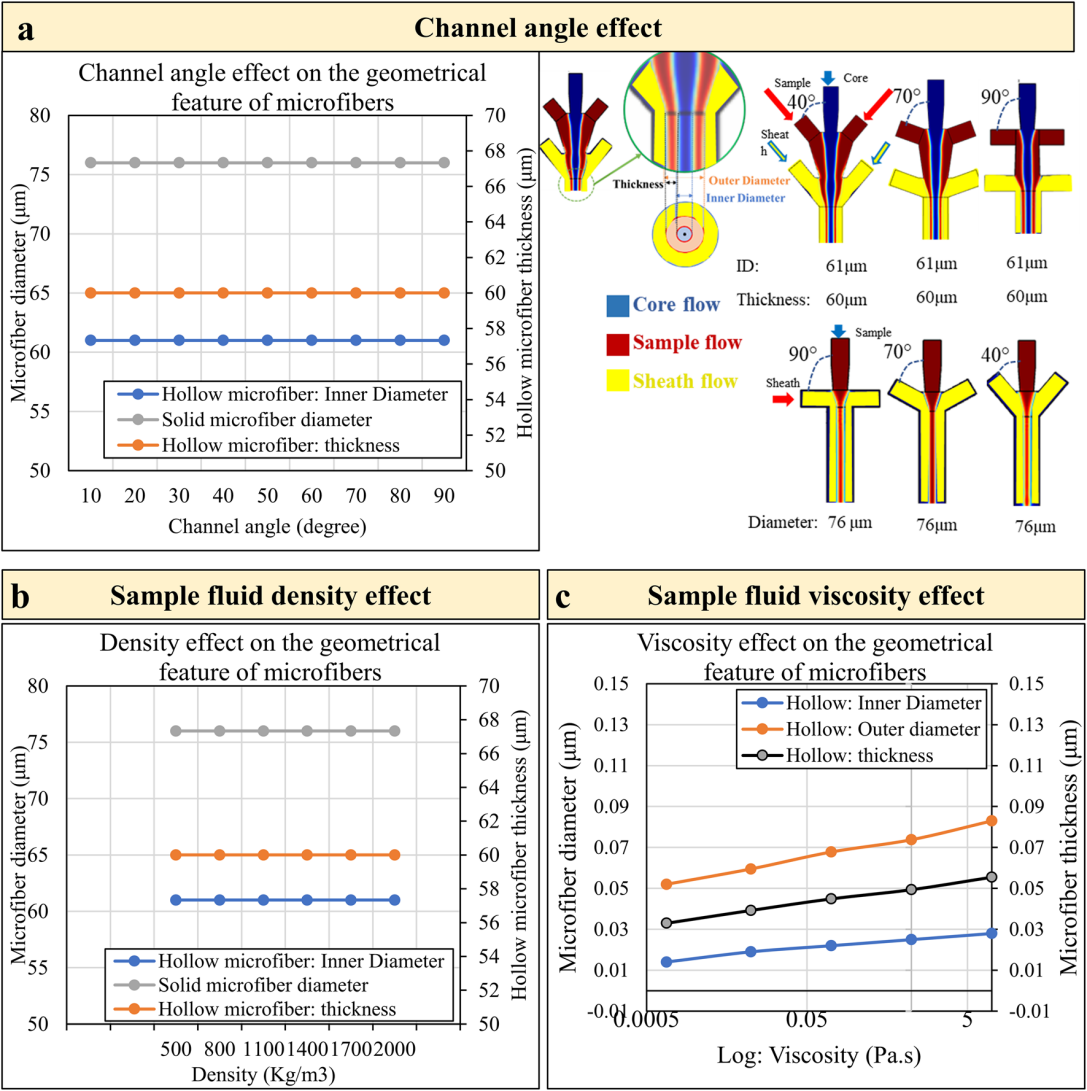
The phase boundary output result of the fabricated (**a**) Left: hollow microfiber for the sample flow (calcium alginate) and sheath flow (CaCl2) is based on different channel angles between 10° to 90° degrees. Right: the phase boundary field of the solid microfluidic channel with different channel angles from 10 to 90 degrees. Herein, for brevity, only the results for 40°, 70°, and 90° are shown. (**b**) The effect of sample fluid density on the geometrical outcomes of microfibers. (**c**) the logarithmic figure of the effect of sample fluid viscosity on the geometrical outcomes of microfibers.

The channel angle is one geometrical parameter that appears to have some effects on the fluid flow and the diameter of the microfiber in the channel. Therefore, it was expected that the larger channel angle of the microfluidic system causes the fluid flows to mix and heavily interact so that the outcome sample flow boundary and consequently the outcome microfiber changes. However, the simulation of the 2D microfluidic channel showed that the channel angle does not have any effect on the inner/outer diameter and thickness of the hollow microfiber and the diameter of the solid microfiber (Fig. 2a). Figure 2 shows the phase boundary output result (blue: core flow, red: sample flow, and yellow: sheath flow) of the fabricated hollow microfiber for the sample flow of sodium alginate and sheath flow of CaCl_2_. It demonstrates that varying channel angles may affect the profile of the hollow fiber on upstream flow but there exists no effect on the geometrical feature size of the fabricated microfiber downstream of the channel. A similar simulation of solid microfiber formation in the microfluidic channel also shows that the diameter of the solid fiber remains constant in the channel angle between 10 to 90 degrees (Fig. 2a). Phase boundary field outputs (red: sample flow and yellow: sheath flow) are illustrated for some of the channel angles in Fig. 2b. Based on Fig. 2, the solid microfiber diameter is invariant with the different channel angles. Therefore, a channel angle of 40° is prescribed for the remaining simulations.

### Density and viscosity effects (Biomaterial type effects)

The density and viscosity of sample fluid are two key parameters within Reynold’s number that can influence fluid flow. Therefore, their effects on the flow boundaries inside the microfluidic channels are investigated to clarify their roles. Based on the numerical results, the sample fluid density in the range from 500 to 2000 kg/m^3^ does not affect the geometrical outcomes of the flow boundaries which leads to the formation of microfiber (Fig. 2b). In contrast, the viscosity of the sample fluid has some effect on the geometrical feature size of the sample flow boundaries. In this respect, as the viscosity increases, the inner diameter, outer diameter, and thickness of the hollow microfiber increase. Figure 2c shows the effect of sample fluid viscosity on the geometrical outcomes of hollow microfibers. Despite the variation of geometrical outcomes by changing viscosity, it has been observed that a big change of viscosity results in small changes in microfiber geometrical feature sizes. Moreover, based on the small channel size in the microfluidics system, low-viscosity fluid is more suitable in comparison with high viscous fluid. Therefore, in this paper, the density and viscosity are to be assumed within the range of low-viscous fluid (Fig. 2).

It is worth noting that the most crucial parameters related to the type of biomaterial that affects the flow of bioink within the channel are density and viscosity. Therefore, these analyses can be considered for other types of bioinks with different densities and viscosity values. Based on the above explanation the density does not any considerable effect on the geometrical shape of the fabricated microfiber. On the other hand, although the viscosity of the biomaterials slightly affects the geometrical features of the structure, this effect is less significant. Moreover, since the viscosity and density parameters have been included in the dimensionless number in Eqs. 8–11, therefore its effect will be included in the CFD simulation. On the other hand, most of the common biomaterials such as alginate, GelMA, alginate-GelMA, etc. that are used in the microfluidic system need to have low viscosity. Therefore, all these biomaterials need to be used in a very low concentration which implies low viscosity. Thus, the viscosity of most of these biomaterial solutions (that are suitable for microfluidic applications) is close to that of the alginate. Despite all this, this paper just claims the accuracy of the model for all biomaterials that have viscosity close to that of alginate. So, a further numerical study is recommended to be done for biomaterials in the future.

### Flow rate effects

The flow rate of the fluids in the microfluidic channels is the major process parameter that affects the structural feature size of the generated hollow and solid microfiber. In the following sections, the flow rate effects on the formation of hollow and solid microfiber are discussed.

#### Hollow microfiber

**Figure 3:**
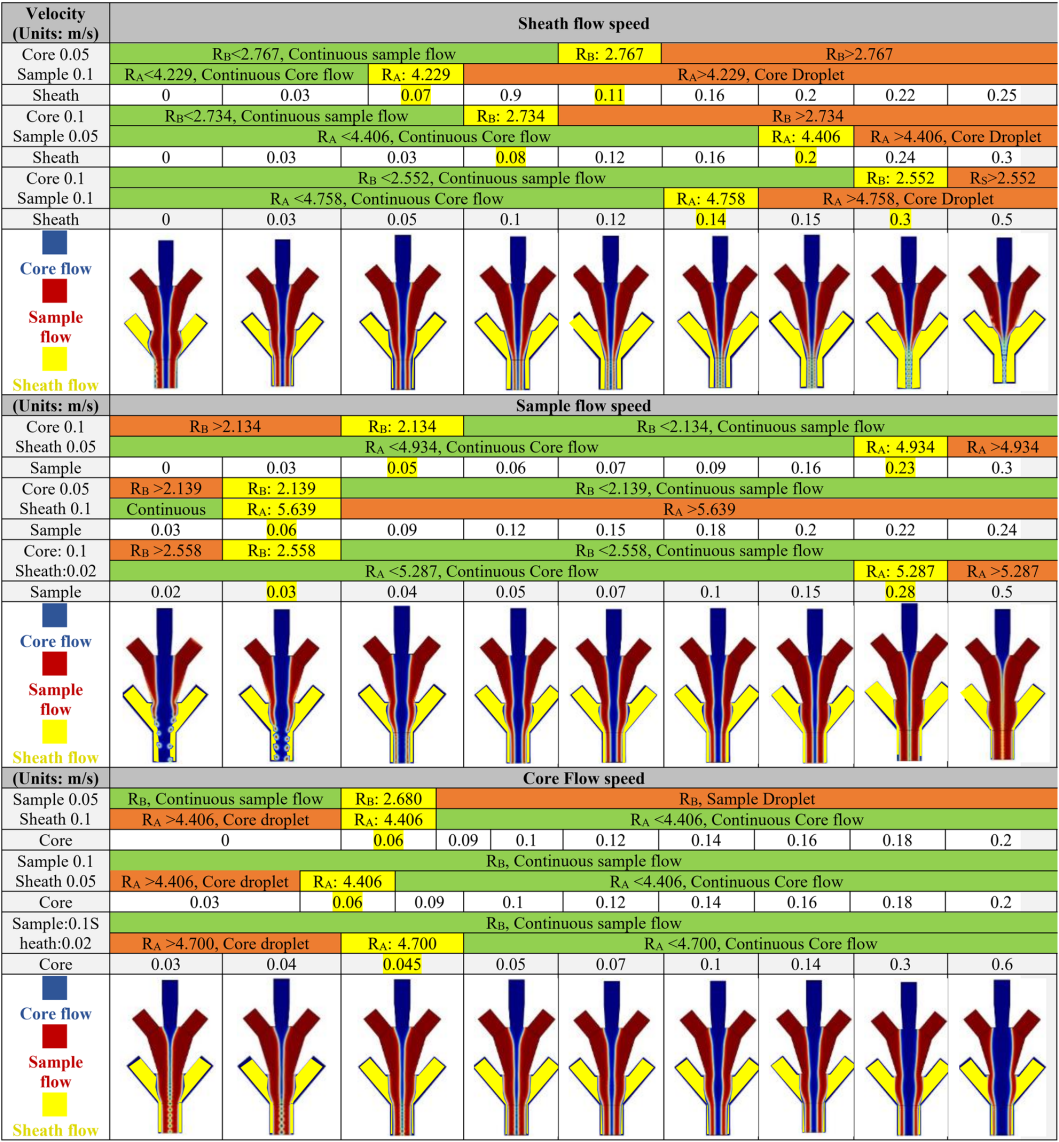
The effect of channel flow rate (Core, Sample, and sheath) on the inner diameter, outer diameter, and wall thickness of the fabricated hollow microfiber using a microfluidic channel. The blue, red, and yellow colors represent the channel A, B, and C. R_A is defined as the ratio of summation of sample and sheath flow Reynold’s number over core flow Reynold’s number. R_B is defined as the ratio of summation of core and sheath flow Reynold’s number over sample flow Reynold’s number. The figures in the section of the sheath, sample, and core flow speed are related to (core 0.1, sample 0.1), (core 0.1, sheath 0.02), and (sample 0.1, sheath 0.02), respectively.

In this section, the microfluidic channel system that led to the hollow fibers will be studied for clarification of the flow rate of the various flows. Generally, the flow rate is the easiest process parameter that can be manipulated to affect the features of the flow boundaries that result in the formation of the microfibers. In Fig. 3, the effects of the different flow rates in channels A, B, and C were examined by keeping the other two channel’s flow rates as a constant value in the microfluidic channel. It is also shown that as the sheath flow rate increases, the flow in channels A and B are moving towards the formation of the droplet. On the other hand, the lower flow rate in channels A and B will lead the flow regime towards droplet formation. After performing the simulation for multiple channel systems with different channel flow rates, a dimensionless number ${R}_{B}$ (Eq. 9) and ${R}_{A}$ (Eq. 10) is found to be useful as a dimensionless parameter to specify the droplet formation regime in the sample fluid and core fluid, respectively. ${R}_{B}$ is defined as the ratio of summation of channel A and B flow Reynold’s number over the Reynold’s number of channel B flow. Likewise, another dimensionless number ${R}_{A}$ is defined as the ratio of summation of sample and sheath flow Reynold’s number over core flow Reynold’s number.

There are several parameters such as viscosity, the flow rate, and channel dimension size that affect the geometrical morphology of the generated microfiber within the microfluidic channel system. Therefore, it is essential to simulate the problem for different values of flow rates. In this regard, a sensitivity analysis is performed to understand the behavior of the flow regime and its correlation towards ${R}_{B}$ and ${R}_{A}$. As Fig. 3 shows, different flow rate magnitude combinations for the core, sample, and sheath flow are modeled and the region of the continuous sample and core flow (green), continuous to droplet flow transforming region (yellow), and droplet-shaped flow region (red) are specified. The upper bound value for the sheath flow rate is prescribed based on the behavior of the microfluidic system in transitioning to the droplet formation. It is observed that a specific value of ${R}_{B}$ and ${R}_{A}$ is found that specify each of these flow regimes. Moreover, the values for ${R}_{B}$ and ${R}_{A}$ that correlate with the transition from continious flow to droplet formation is independent of the channel flow rate values. One major advantage of ${R}_{B}$ and ${R}_{A}$ is the fact that they are based on well-known dimensionless Reynold’s number which contains viscosity, velocity, characteristic diameter, and density. Therefore, ${R}_{B}$ and ${R}_{A}$ are independent of these properties. However, here the results for sodium alginate as a sample fluid and CaCl_2_ as core and sheath fluid are verified. Therefore, these ratios can be used for different magnitudes of flow rate and channel diameter. These sets of numerical simulations are performed for various values of the flow rate in the channel to ensure that the value of ${R}_{B}$ and ${R}_{A}$ in which the flow transition happens is kept constant. Therefore, regardless of the value of the flow rate for flow in the channels, there is a constant ${R}_{B}$ and ${R}_{A}$ value that specifies the transition of the sample flow boundaries from continuous to droplet form. However, the transitional value of ${R}_{A}$ and ${R}_{B}$ is slighly different for different values of the channel flow rate, observations that can be attributed to numerical calculation errors. Furthermore, an average value of this transitional value has been calculated to distinguish the droplet region versus continuous sample flow. So, based on the simulation it can be implied that:

- The ${R}_{B}$ more than 2.51 ± 0.27 results in droplet formation in the sample flow
- The ${R}_{B}$ less than 2.51 ± 0.27 results in the continuous sample flow
- The ${R}_{A}$ more than 4.76 ± 0.50 results in droplet formation in core flow
- The ${R}_{A}$ less than 4.76 ± 0.50 results in continuous core flow

While the modeling reveals that the core and sample flow may transfer to a droplet shape, it is difficult to observe the formation of a droplet in the sheath flow. This phenomenon may be due to the surface tension between sheath flow and the microfluidic channel wall and the no-slip condition in these walls that make it difficult for the fluid to disconnect. It is noteworthy that the change in the sheath flow rate does not have a significant effect on the thickness and diameter of the fabricated hollow fiber. This is owing to the fact that this channel is typically used to provide lubrication or crosslinking, so higher flow rates are not typically prescribed. On the contrary, the change in the flow rate of the sample and sheath flow rate can significantly influence the geometrical properties of the microfibers. Therefore, it is shown that the ratio of the flow Reynold’s number (${R}_{B}$ and ${R}_{A}$) in the channel A, B, and C is the most prominent factor that can control the inner/outer diameter and thickness of the fabricated hollow microfiber.

**Figure 4:**
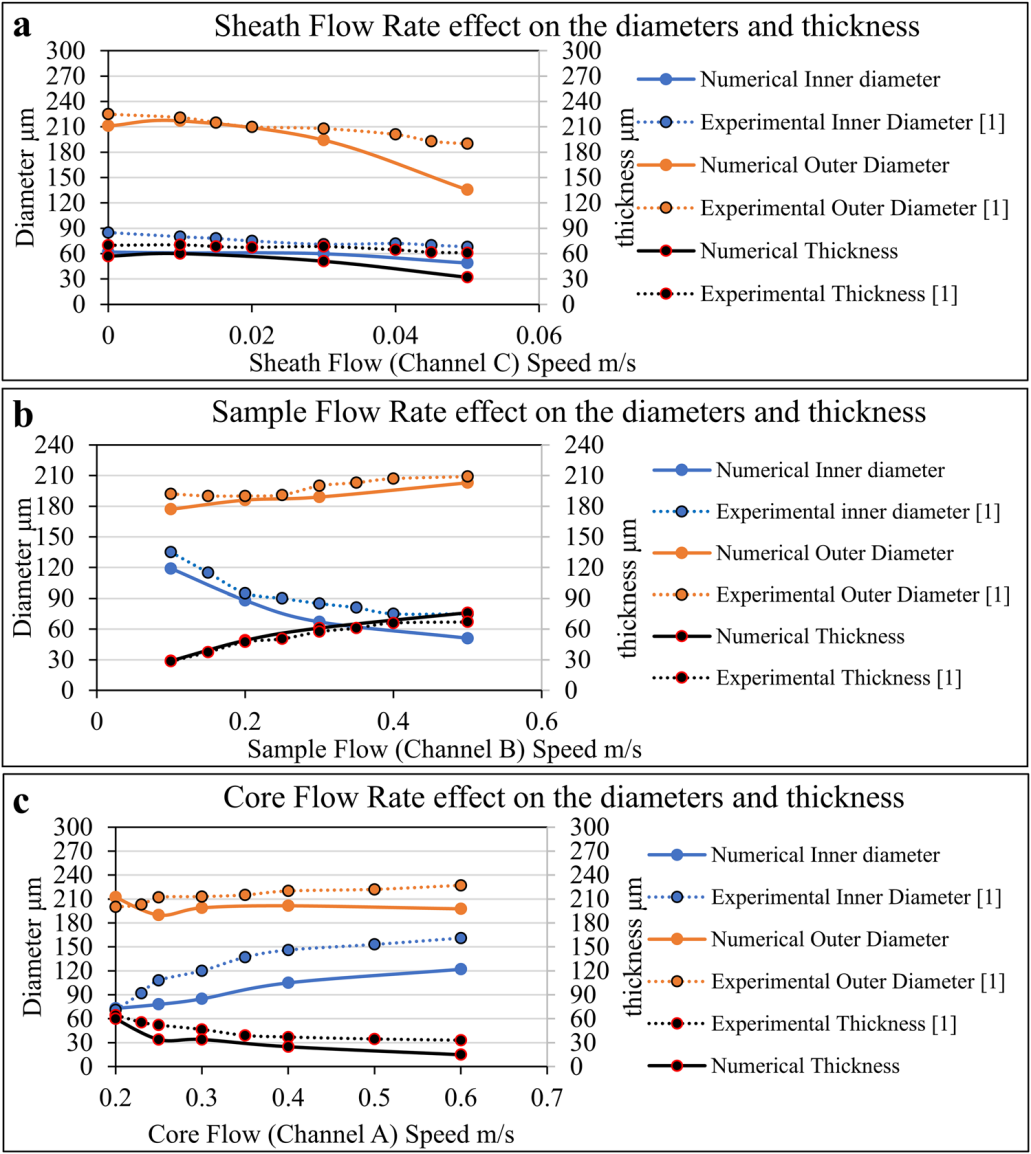
Experimental results of the hollow microfiber inner/outer diameters and thickness for (**a**) sheath flow effect, (**b**) sample flow effect, and (**c**) core flow effect^1^.

**Figure 5:**
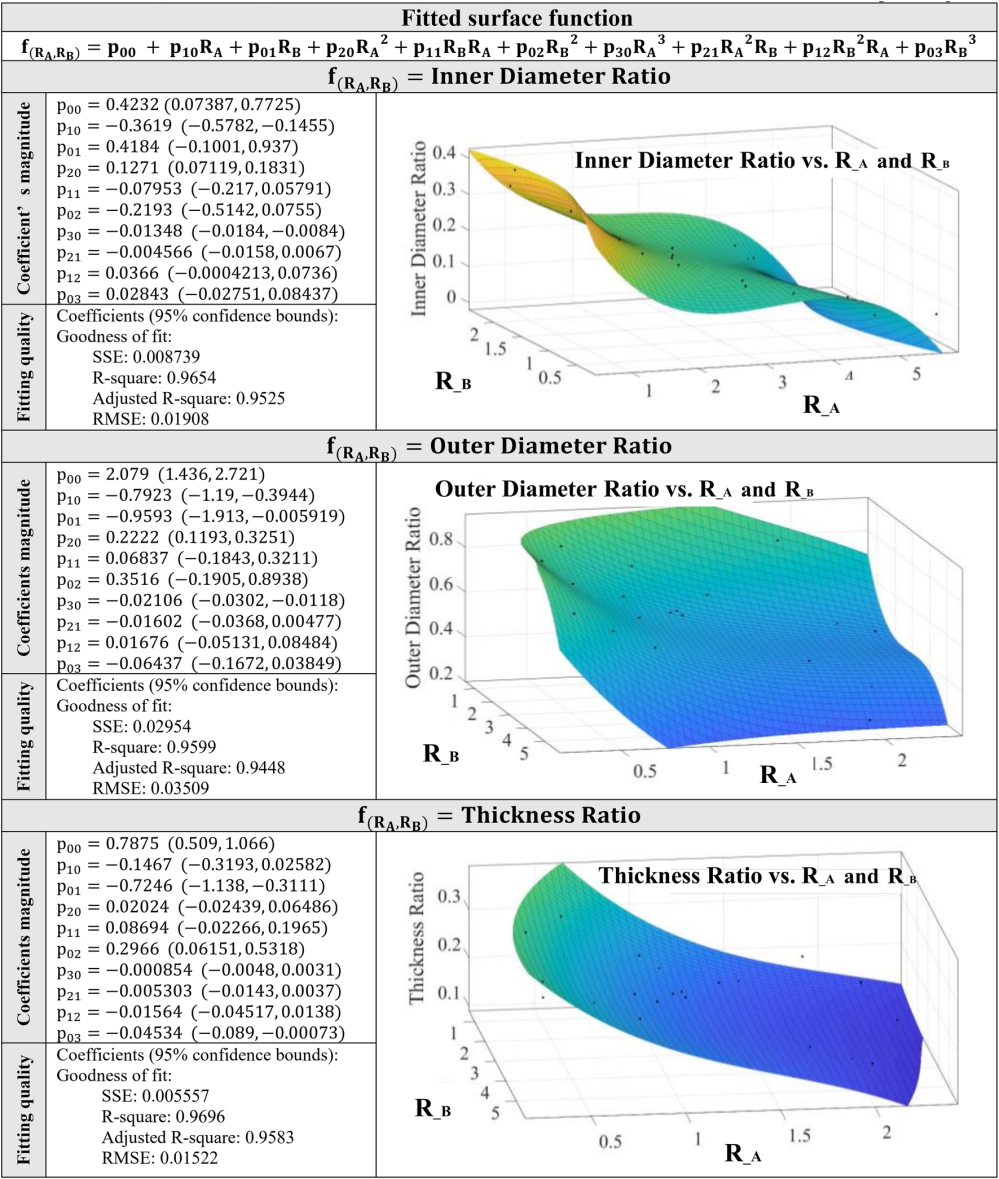
The prediction equation of inner/outer diameter ratio and wall thickness ratio based on ${{\varvec{R}}}_{B}$ and ${{\varvec{R}}}_{A}$.

In this paper, the inner diameter, outer diameter, and wall thickness of hollow microfiber results are compared to the experimental data in Figs. 4a, 5b,c for core, sample, and sheath flow rate analysis and the numerical model results were verified^1^. The trends of the change of inner/outer diameter and thickness in the numerical and experimental models are the same. However, the value slightly diverges, which may be due to the uncertainties of either experiment results or numerical solution simplifications such as 2D modeling of the microfluidic channel system^1^. Moreover, in most of the cases, the experimental data shows a higher value of microfiber inner/outer diameter and thickness compared to that yielded by the numerical results. The numerical value of microfiber feature size is 0 to 28.6%, 15.4 to 27.9%, and 14.9 to 47.5% lower than experimental data for the outer diameter, inner diameter, and thickness of the microfiber in Fig. 4a. Regarding the data of sample flow rate effect, the numerical value of microfiber feature size is 2.9 to 7.8%, 7.3 to 32.0%, and − 1.7 to − 13.4% lower than experimental data for the outer diameter, inner diameter, and thickness of the microfiber in Fig. 4b. Lastly, the numerical value of microfiber feature size is − 6.1 to 12.9%, − 2.8 to 29.1%, and 6.9 to 54.5% lower than experimental data for the outer diameter, inner diameter, and thickness of the microfiber in Fig. 4c. The difference between the experimental and numerical data is observed to increase as the flow speed increases.

The main advantage of numerical simulation is that it serves as a surrogate for experimental approaches. Therefore, in Fig. 5, a predictive equation is introduced to estimate the inner diameter ratio, outer diameter ratio, and wall thickness ratio of the fabricated hollow microfiber using MBP without initiating any experimental process parameter tuning. Thus, by using this approximating formula, the geometrical dimension of the hollow microfiber based on the targeted values for ${R}_{B}$ and ${R}_{A}$ is predetermined. This formulation, which is derived by fitting a curvature surface into the numerical modeling data, is independent of any specific channel size and flow rate due to its inherent dimensionless base. This formulation is based on polynomial function fitting of 3rd order. The diameter and wall thickness ratio which is the ratio of microfiber diameter or wall thickness to the outlet channel diameter is chosen because the ${R}_{B}$ is a dimensionless number so that the diameter parameter needs to be generalized. Therefore, the diameter ratio is also a dimensionless number that can be used for a variety of channel sizes. It is necessary to mention that the formulation in Fig. 5 is applicable for the microfluidic flow system with ${R}_{B}$ less than 2.51 ± 0.27 and ${R}_{A}$ less than 4.76 ± 0.50. This is because droplet formation is observed when ${R}_{A}$ and ${R}_{B}$ are greater than aforementioned values. Since the numerical model has been verified by the available experimental data in the literature, the proposed formulation in Fig. 5 returns valid results.

While the numerically derived geometrical features of the hollow microfibers showed an agreement with the experimental data in reference^1^, the predictive model needs to be validated with another experimental result. Therefore, the geometrical features such as inner diameter, outer diameter, and thickness that were calculated from the predictive equation in Fig. 5b, were compared with the experimental derived geometrical features of hollow calcium alginate microfibers fabricated with a double co-axial flow capillary microfluidic device^22^. The results showed an agreement between the predicted values and the experimentally measured values. However, there is some difference between these results with is ascribed to the complexity of the fluid dynamics in the three-dimensional experiment.

To show that the fabricated microfiber geometry is independent of the value of flow rates (not the ratio of flow rates), the same model is simulated for different values of flow rate with the same ratio. This figure shows that the inner diameter, outer diameter, and wall thickness of the hollow microfiber do not change with the different flow rate magnitude (in the constant ratio).

#### Solid microfiber

**Figure 6:**
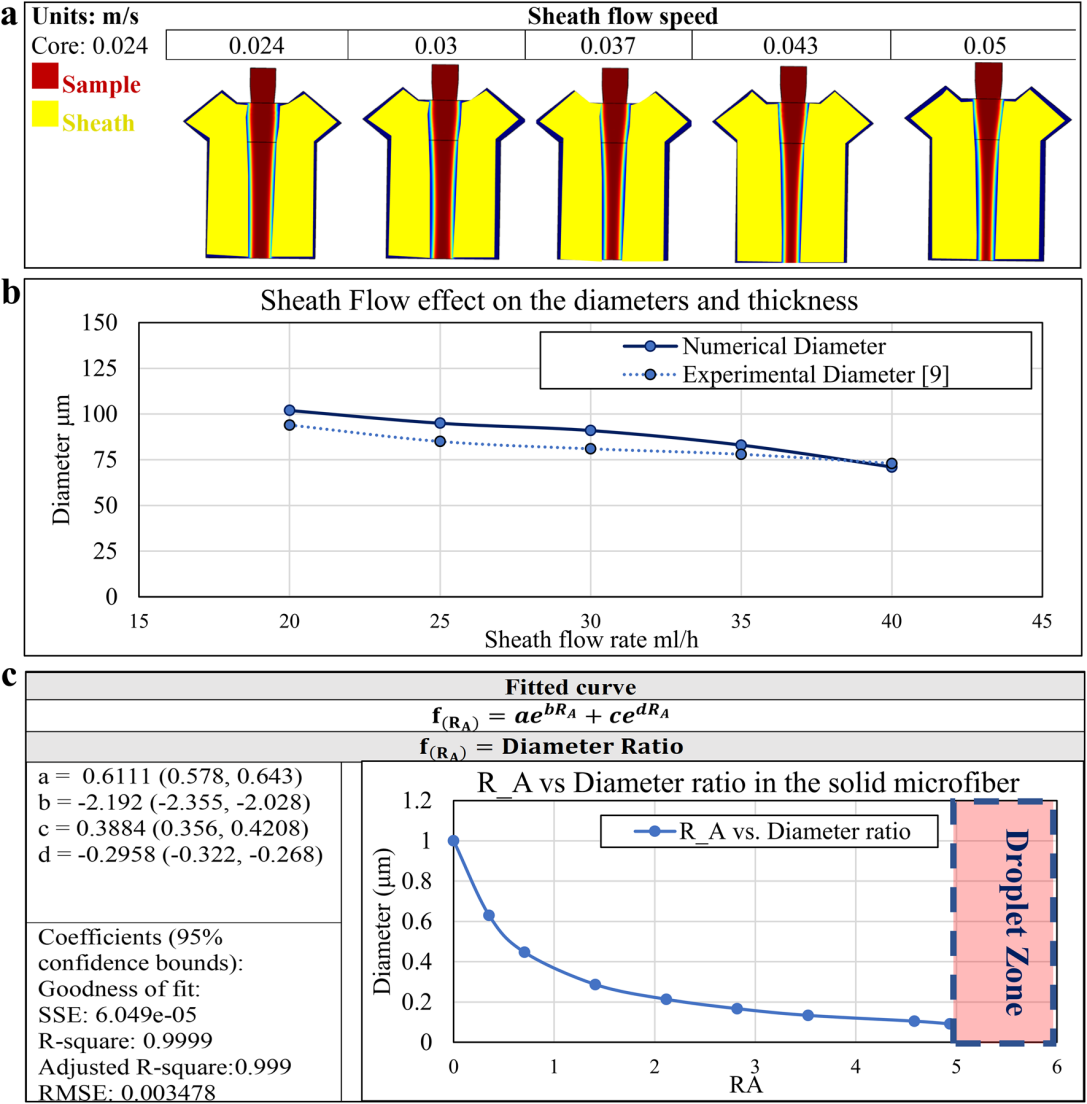
Solid microfiber generation using MBP (**a**) The phase boundary field output of the calcium alginate solid microfiber fabricated inside the microfluidic channel based on different sheath flow rate, (**b**) The comparison of the experimental and numerical diameter of the solid microfiber3 (**c**) The prediction equation of solid diameter ratio and wall thickness ratio based on ${{\varvec{R}}}_{B}$ and ${{\varvec{R}}}_{A}$.

Although the microfluidic system design for the fabrication of hollow versus solid microfiber appears to be different, there exist identifiable similarities. If channel C of the hollow microfiber is blocked, then the result would be a microfluidic system that is capable of the fabrication of solid microfiber. Akin to the simulation for the hollow microfiber, the flow rate plays a significant role in controlling the geometrical properties of the fabricated solid microfiber. Figure 6a shows the phase boundary field output of the solid microfiber fabricated inside the microfluidic channel. Herein, the ratio of the sheath flow rate to the sample flow rate is observed to be directly related to the diameter of the solid microfiber. Besides, the viscosity and channel diameter have a significant effect on the diameter of solid microfiber. Furthermore, based on the comparison between the solid microfiber diameters output in the numerical simulation and the experimental diameter values of calcium alginate^3^ in Fig. 6b, the magnitude and the decreasing trend of the microfiber diameter are the same. This figure shows that the error value of numerical and experimental values is less than 10.5%. Therefore, the numerical model accuracy is acceptable while it can be improved.

So, like the hollow microfiber, a dimensionless number ${R}_{A}$ which is defined as the ratio of channel B flow Reynold’s number over channel A flow Reynold’s number, is a criterion that specifies the droplet formation regime in the sample fluid. Also, this variable can be leveraged to correlate the diameter of fabricated microfiber and process parameters such as viscosity, channel size, density, and flow rate of the fluid in channels B and C. The only parameter that affects the diameter is ${R}_{A}$. Again, here a useful formula is introduced that relates ${R}_{A}$ to the solid microfiber diameter ratio. The diameter ratio which is the ratio of microfiber diameter to the outlet channel diameter is chosen because the parameter ${R}_{A}$ is a dimensionless number, so that the diameter parameter needs to be generalized. Therefore, the diameter ratio is also a dimensionless number that can be used for a variety of flow rates and channel sizes. This formulation is an exponential based function (see Fig. 6c) that relates the ${R}_{A}$ to the solid microfiber diameter ratio (dimensionless). Similar to the microfluidic channel for the fabrication of hollow microfiber, there is a region of continuous sample flow, continuous to droplet sample flow, and droplet flow region in the microfluidic channel for the fabrication of solid microfiber. Here, the dimensionless number ${R}_{A}$ assumes a critical value of approximately 5.14. Therefore, for ${R}_{A}$ a value higher than 5.14, the droplet regime appears. On the other hand, the ${R}_{A}$ value lower than 5.14 results in a continuous solid microfiber. It is noteworthy to mention that the predictive formulation in Fig. 6 is for microfluidic flow with the ${R}_{A}$ value lower than 5.14. Since the simulation of different channel geometries in Fig. 3 yields an approximately identical transitional ${R}_{A}$ value, therefore, the critical droplet formation ratio is independent of the channel geometry as well.

#### Droplet generation

**Figure 7:**
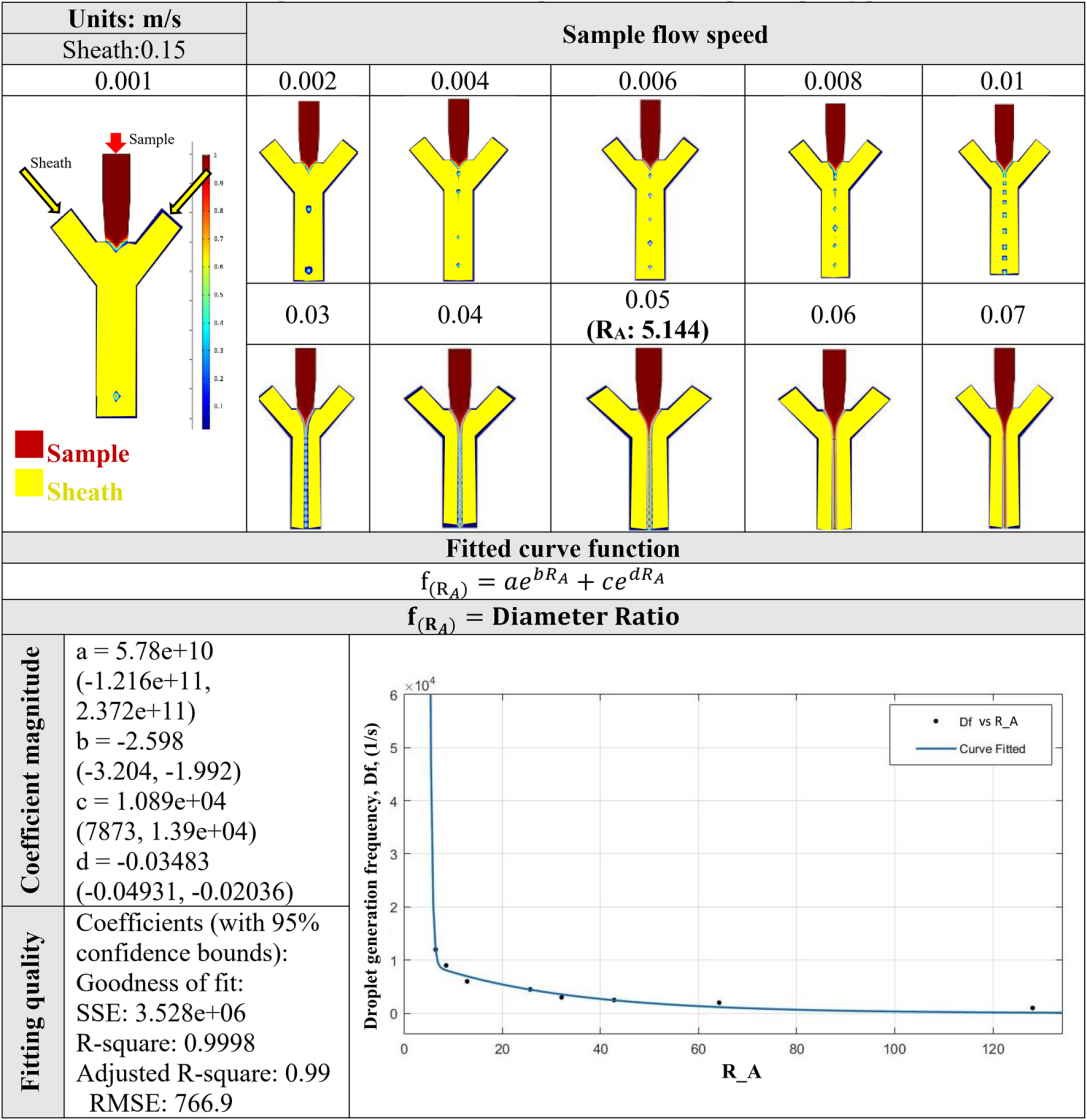
The droplet formation of calcium alginate and microdroplet frequency prediction.

One important aspect of the fluid flow in the microfluidic channel is the possibility of droplet generation for a prescribed condition. In the microfluidic channel, for the critical ${R}_{A}$ value of 5.14 the flow starts to transition from continuous flow into a droplet regime which means that there is an infinite number of droplets that are connected. As the flow regime proceeds and ${R}_{A}$ value begins to increase to the higher values, the distance between consecutive droplets increases. This means that the rate of droplet generation increases. In general, the simulation of such a microfluidic channel demonstrates that the higher the ${R}_{A}$ value is, the lower the frequency of droplet generation is. Figure 7 shows the droplet formation of sample flow for the variety of sample flow rate speeds. Also, a straightforward formula has been introduced to relate the ${R}_{A}$ to the frequency of droplet generation. This formulation is based on the exponential function. In the condition when ${R}_{A}$ value is 5.14 (transitional value), a vertical asymptote is seen in Fig. 7. These simple and practical formulation is a great way to reduce the cost of experimental investigations because the researchers in the field can simply calculate the ${R}_{A}$ in case of solid microfiber or ${R}_{A}$ and ${R}_{B}$ in case of hollow microfiber to predict the geometrical dimension of the final solid and hollow microfiber, respectively.

## Ongoing and future work

Microfluidic technology has opened a new avenue in the field of bioprinting and additive biomanufacturing towards the fabrication of precision, complex tissue constructs. Therefore, combining the microfluidic concept with traditional bioprinting systems yields a microfluidic-based printhead that enhances engineered tissue properties with higher controllability over the geometrical outcomes of the tissue constructs. Moreover, the microfluidic-based printhead opens up an opportunity for researchers to add more modules to the printhead to increase their control over the final engineered tissue. This add-on module could be a mixing unit or crosslinking unit which results in the fabrication of functionally graded filaments in terms of polymer and/or cell concentration within the bioink. Above all, although the numerical model that has been discussed in this paper has been verified using the experimental data in the literature (Fig. 6b), these numerical models and data need to be validated with the additional experimental investigation.

**Figure 8:**
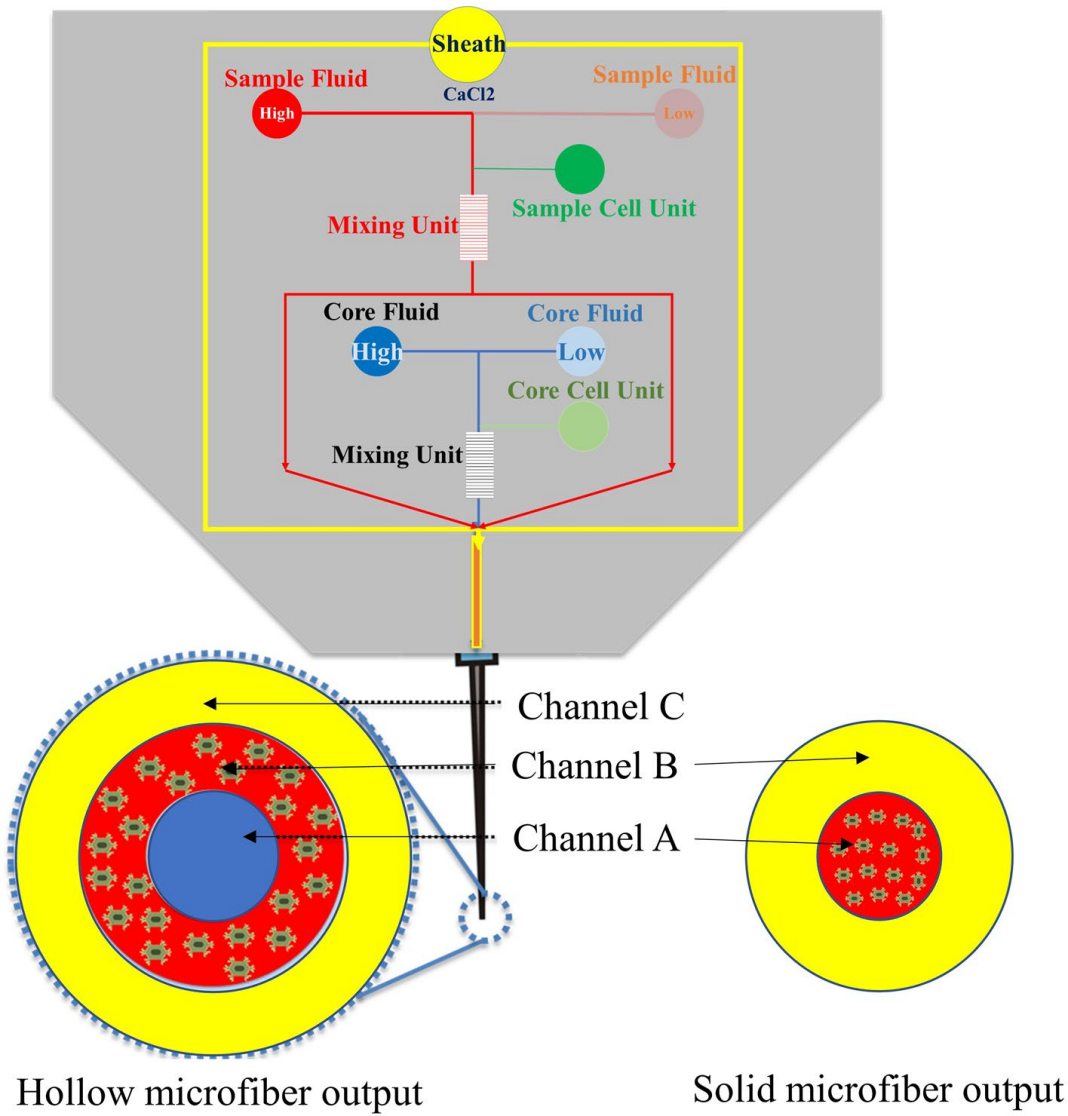
The microfluidic-based bioprinter system, (**a**) channel system design of the microfluidic printhead, (**b**) the whole bioprinting system set-up.

From the perspective of the future design and research, the concept is advanced herein for a microfluidic printhead (Fig. 8a) capable of fabrication of functionally graded tissue constructs in terms of filament geometrical feature size, filament shape (hollow and solid microfiber), multi-material filaments, and variety of polymer/cell concentration. In addition, the channel inlets could be opened or closed so that the practictioners will be able to fabricate solid and hollow microfibers. Moreover, instead of the crosslinking fluid, a secondary bioink may be used so that a multimaterial filament can be fabricated. Moreover, there is a need to study the effect of cell particles inside the fluid flow in the microfluidic channel system which needs to be clarified.

After the numerical simulation, the MBP can be made using different fabrication methods such as references^23–25^ (e.g., 3D printing, PDMS molding, and Micromachining). Then, the MBP should be connected to the bioprinting system (Fig. 8b). For the bioprinter system configuration, a pressure-driven system is designed using a combination of pressurized air, pneumatic valves, syringes, and medical tubing. Flow controllers should be connected to the pressurized air to provide the desired input pressure. In Fig. 8a, the schematic design of the microfluidic-based bioprinter has been shown which will be discussed in future works. However, the current paper's scope is on the design, modeling, and characterization of the fabricated hollow and solid microfibers.

## Conclusions

In this investigation, the design and numerical evaluation of a microfluidic-based printhead are implemented to enable controlled microfiber extrusion for bioprinting functionally graded tissue constructs. Based on the simulations, the geometrical dimensions for both hollow and solid microfiber fabricated using a microfluidic channel system is dependent on the dimensionless parameters ${R}_{A}$ and ${R}_{B}$ which are independent of density, viscosity, channel diameter size, and fluid flow rate. In general, it is found that when ${R}_{A}$ exceeds 4.76 ± 0.50, the core flow will overlap with a droplet formation regime, and for ${R}_{A}$ lower than 4.76 ± 0.50, a continuous flow will prevail. Also, if ${R}_{B}$ value exceeds 2.51 ± 0.27 the sample flow will form a droplet and for ${R}_{B}$ lower than 2.55 a continuous flow will appear. Also, a simple and practical mathematical formula that correlates the parameters ${R}_{A}$ and ${R}_{B}$ with dimensionless geometrical dimensions is presented which, regardless of channel size and flow rate value, can be leveraged to predict the approximated value of diameters and wall thickness. This formula is valid for the prescribed range of fluid density and hydrogel material viscosity values studied. The outcome of the studied microfluidic-based printhead system is the ability to establish the permissible window of design, bioink material, and process parameters that enables the fabrication of functionally graded tissue constructs. The systematic analyses presented herein will provide microfluidic-based bioprinting practitioners with the insight to enhance the quantitative aspects of printing process optimization^26^.
